# Retraction: MicroRNA-217 Regulates WASF3 Expression and Suppresses Tumor Growth and Metastasis in Osteosarcoma

**DOI:** 10.1371/journal.pone.0269901

**Published:** 2022-06-08

**Authors:** The PLOS ONE Editors

Following the publication of this article [1], similarities were noted between this article and articles submitted by other research groups, including [2–10], of which one article [10] was previously retracted [11].

Similarities included the following figures, which appear to fully or partially overlap, despite being published in different articles and representing different conditions:

- untreated and scramble panels in Figure 2D of [1], and scramble panel in Figure 2C of [2].
- miR-217-mimic panel in Figure 2D of [1], and inhibitor panel in Figure 3E of [6].
- pcDNA + scramble panel in Figure 4E of [1], and control panel in Figure 3E of [9].
- pcDNA-WASF3 + scramble panel in Figure 4E of [1], and miR-561-inhibitor panel in Figure 3E of [9].
- WASF3 panel in Figure 3D of [1], L1CAM panel in Figure 4D of [4], PAQR3 panel in Figure 4D of [6], and BRD7 panel in Figure 4C of [7].

Although the corresponding author initially replied to acknowledge receipt of our message, *PLOS ONE* did not receive responses to the queries regarding these concerns by the end of the original deadline and extension.

The unresolved concerns call into question the validity and provenance of the reported results, and the adherence of this article to the PLOS Authorship policy. Therefore, the *PLOS ONE* Editors retract this article [1].

All authors did not comment on the retraction decision, did not respond directly or could not be reached.
